# Do the Pre-Existing Class III and Class V Composite Restorations Affect the Sealing Ability and Integrity of 3D-Printed Laminate Veneer Margins? An In Vitro Study

**DOI:** 10.3390/jfb17050249

**Published:** 2026-05-17

**Authors:** Abdulkhaleq Mohammed Qaraghuli, Edoardo Ferrari Cagidiaco, Marco Ferrari

**Affiliations:** Department of Medical Biotechnologies, School of Dentistry, Division of Fixed Prosthodontics, University of Siena, 53100 Siena, Italy; marco.ferrari@unisi.it

**Keywords:** three-dimensional printing, dental veneers, composite resins, microleakage, dental marginal adaptation, resin cements, anterior teeth, class III restoration, class V restoration, digital dentistry

## Abstract

**Background:** The application of veneer restorations over previously composite-restored anterior teeth presents significant clinical challenges, particularly in achieving optimal marginal sealing. **Aim:** This in vitro study aimed to evaluate the marginal integrity and sealing ability of different 3D-printed resin veneer restorations on sound versus composite-restored anterior teeth. **Materials and Methods:** Eighty freshly extracted human anterior teeth (40 central incisors and 40 canines) were randomly assigned into two main groups: sound teeth and composite-restored teeth. All the teeth received 3D-printed resin veneer restoration utilizing two different types of 3D-printed resin (GC Temp Print, GC, Tokyo, Japan; and Varseosmile Triniq, BEGO GmbH & Co., Bremen, Germany). The specimens were then subjected to microleakage, marginal fitness, cement void, and cement loss testing. **Results:** There were no statistically significant differences among all examined groups. Microleakage scores were predominantly 0 across all groups, with median values of 0 at both cervical and proximal surfaces. Marginal fitness showed fit percentages ranging from 20% to 100%, while cement voids and cement loss were rare events (<10%). Statistical analysis confirmed no significant differences between groups (*p* > 0.05), with *p*-values ranging from 0.151 to 1.000. **Conclusions:** No, the presence of pre-existing composite restorations did not adversely affect 3D-printed veneer performance. The marginal integrity and sealing ability of two different 3D-printed resin veneers are not affected by the presence of previous composite restoration on cervical and proximal surfaces for both incisor and canine teeth. 3D-printed veneers applied to sound and restored teeth showed good marginal integrity and proper sealing ability.

## 1. Introduction

Veneer restoration over restored teeth with composite resin filling represents a significant challenge regarding the durability and success of such a type of treatment [1].

In anterior teeth, composite restorations can be located on cervical and proximal surfaces such as CL V and CL III [2]. Dental veneers have revolutionized aesthetic and restorative dentistry by providing a minimally invasive solution for improving the appearance and functionality of teeth and are among the most frequently utilized treatments in aesthetic dentistry because of their biocompatibility, durability, and aesthetic qualities [3].

Nevertheless, the challenges associated with veneer restoration over previously restored teeth are obvious when the veneer restoration margin is positioned on the composite surface [4]. Commercially available veneer restoration materials possess mechanical and physical properties that do not always align with those of composite resins. The mismatch between veneer and composite resin materials leads to several problems that undermine the integrity of the veneer restoration margin in terms of surface longevity [5]. For instance, a key factor in the failure and/or lifespan of veneer restoration is the difference in the modulus of elasticity and coefficient of thermal expansion between the composite filling and the veneer restoration material [6].

Veneer restorations made from materials that act similarly to filling materials, such as resin-based veneer restorative materials, may improve the effectiveness of veneer restoration treatments [7]. Additionally, a newly available 3D-printed resin for the fabrication of indirect veneer restorations has been introduced to the market, which may improve the quality and predictability of such treatments.

The rapid and continuous advancement of additive manufacturing in recent years has opened new possibilities in esthetic and restorative dentistry. 3D-printing technologies such as digital light processing (DLP), stereolithography (SLA), and liquid crystal display (LCD)-based printing demonstrated superior precision and surface resolution in the production of indirect dental restorations. Furthermore, modern techniques such as digital light processing (DLP) allow for the fabrication of complicated geometries and multi-material structures with enhanced precision. Recently, the advances in high-resolution printing and multi-material fabrication have further increased the clinical uses of 3D printing and facilitated the development of specific and customized patient dental treatments. These advancements show the huge potential of additive manufacturing technology to improve the performance and clinical results of indirect restorations [8,9,10].

Although dental veneer restorations over composite substrates have gained increasing attention, most available studies that have been conducted have mainly focused on tooth structures that are still intact or on conventional ceramic or milled veneer materials. There is still a limited amount of evidence concerning the behavior of newly introduced 3D-printed resin materials when applied over previously restored composite surfaces, particularly in terms of marginal adaptation and sealing performance. Furthermore, there is a lack of comparative data that evaluates the interaction between various 3D-printed resin materials and the composite restoration that lies beneath them. This highlights a clear gap in the existing literature regarding the laboratory and clinical performance of these materials.

The laboratory investigations that are frequently employed to assess the effectiveness of veneer restoration include microleakage testing, cement void evaluation, assessment of cement gaps, and marginal fit evaluation. Also, depending on modified United States Public Health Service (USPHS) parameters, a recent study showed that the survival rate of 3D-printed resin (Temp Print, GC Corp, Tokyo, Japan) was 100% at the one-year recall [11].

Research on veneer restoration utilizing freshly extracted anterior teeth offers conditions that more accurately reflect the behavior of restoration samples on natural teeth. The shape, size, and mechanical and physical properties of freshly extracted teeth create superior testing conditions compared to typodont models.

The aim of this study is to evaluate the veneer restoration over composite-restored teeth using different 3D-printed resin materials. The null hypothesis is that pre-existing composite restorations on anterior teeth do not affect the marginal integrity and sealing ability of 3D-printed veneer restorations.

## 2. Materials and Methods

### 2.1. Sample Size Calculation

The sample size was calculated using G*Power (version 3.1.9.7; developed by Franz Faul, University of Kiel, Germany). Based on a two-tailed test, an alpha error probability of 0.05, a statistical power of 95%, and a large effect size of Cohen’s D of 0.85, a sample size of 80 was determined. As such, 10 specimens per subgroup were considered sufficient to detect statistically significant differences [12].

### 2.2. Sample Selection and Inclusion Criteria

A total of eighty sound, freshly extracted human anterior teeth (40 central incisors (I) and 40 canines (C)) were collected. Teeth were extracted for reasons unrelated to this study. After extraction, all teeth were cleaned of soft tissue debris and calculus using hand instruments and stored in normal saline solution at room temperature to prevent dehydration and preserve their physical properties until use. The storage medium was periodically refreshed to avoid contamination, and all teeth were used within 1–3 months of extraction.

The teeth included in the study were intact and free from caries, cracks or fractures and structural defects. Teeth with restorations, fractures, severe wear or previous endodontic treatment were excluded.

### 2.3. Grouping and Randomization

Samples were randomly allocated using a computer-generated randomization method into two main groups:

- Sound teeth (20 sound central incisors (SI) and 20 sound canines (SC)).
- Composite-restored teeth. Composite restorations (Class III and Class V) were performed on half of the samples (20 central incisors (RI) and 20 canines (RC)) using a standardized protocol. Each main group was further subdivided according to:
- Tooth type (incisor/canine);
- Veneer material (Temp print/Varseosmile triniq).

**Figure 1:**
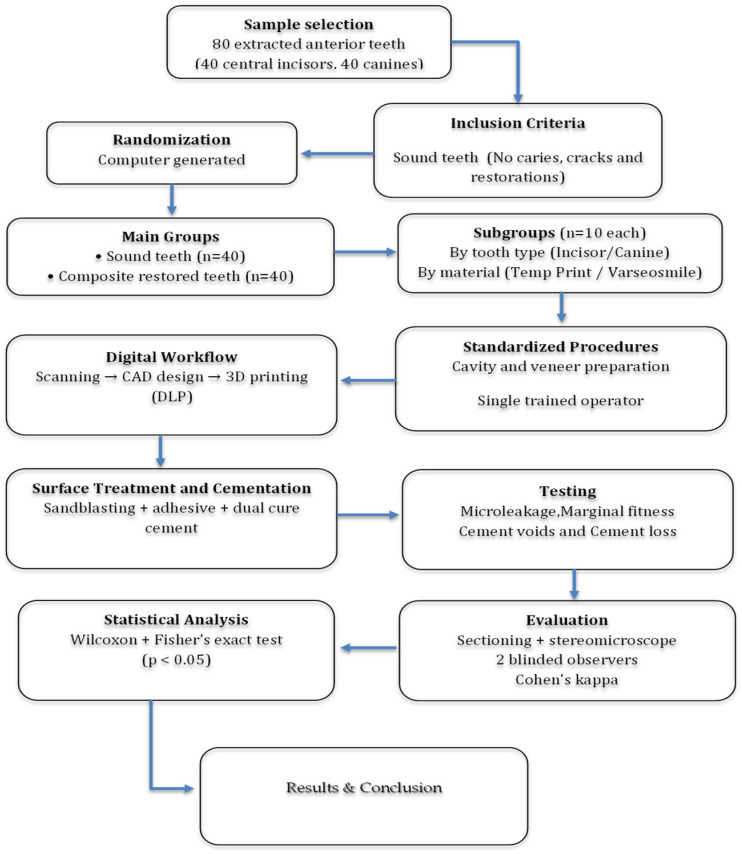
Flowchart of the study design, sample grouping and experimental procedures.

This resulted in eight subgroups (*n* = 10 per group). The study design and grouping of samples are illustrated in Figure 1.

### 2.4. Standardization and Confounding Control

To minimize variability and control confounding factors, all procedures, including cavity and teeth preparation, composite restorations, veneer fabrication, and cementation, were performed by a single experienced operator with training in fixed prosthodontics and restorative dentistry. Furthermore, Class III and Class V cavities were prepared using standardized dimensions to ensure consistency across specimens. Class III cavities were prepared with approximately 2 mm inciso-gingival height, 2 mm bucco-lingual width, and 1 mm depth. This standardized approach is consistent with previously published methodologies [13,14].

**Figure 2:**
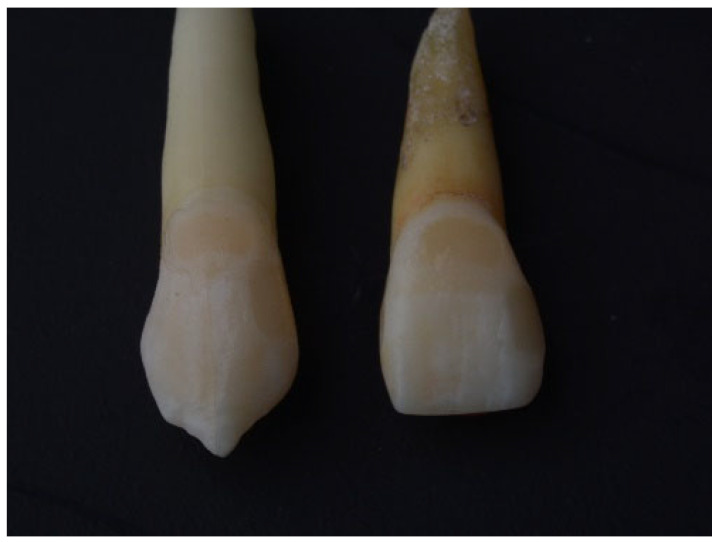
Class III and Class V composite restorations.

Class V cavities were standardized to approximately 3 mm mesio-distally (width), 2 mm occluso-gingivally (height), and 1.5 mm depth at the cemento-enamel junction [15]. All preparations were performed using calibrated instruments and verified with a periodontal probe. A new bur was used after every five preparations to ensure cutting efficiency. Class III and V cavities were filled with composite restorations after appropriate adhesive protocols for the tooth surface, as shown in (Figure 2). All procedures were conducted under magnification.

### 2.5. Veneer Preparation

**Figure 3:**
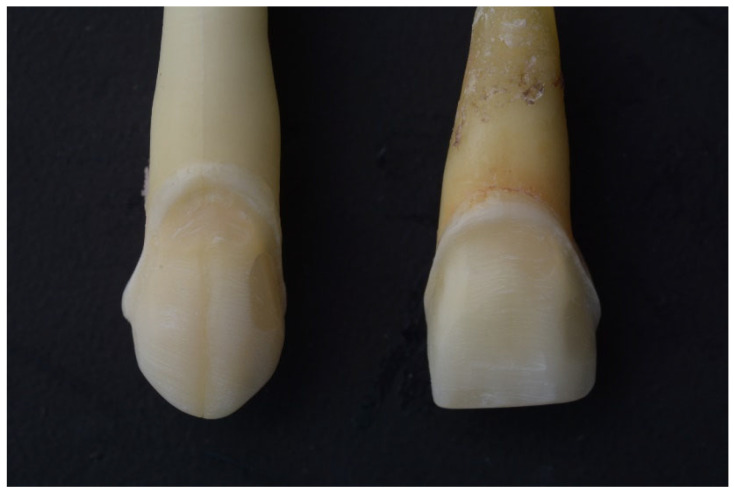
Veneer preparation on the labial surface of restored teeth.

Veneer preparation was done on all 80 teeth using a round-end tapered diamond bur to create a light chamfer finish line. The preparation was kept within the enamel as much as possible to enhance the adhesive bonding; as such, the margins of the 3D-printed veneer restoration were positioned on the enamel of the sound teeth group and partially on the composite surface of the restored teeth group [16]. This procedure was aided using magnification loupes, and final surface finishing utilized fine-grit burs and flexible disks for smoothness [17], as shown in (Figure 3).

### 2.6. Digital Workflow and Fabrication

**Figure 4:**
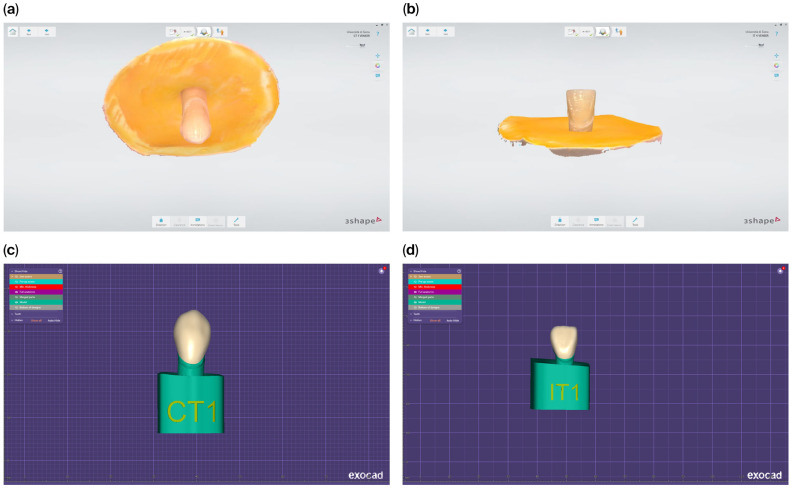
Digital workflow: (**a**,**b**) Digital scanning of prepared teeth; (**c**,**d**) veneer design of samples (CAD software).

Digital scanning of prepared teeth for veneer fabrication was performed using a high-resolution intraoral scanner (3Shape, Copenhagen, Denmark). Veneers were designed using CAD software, exocad DentalCAD (version 2.3 Elefsina, exocad GmbH, Darmstadt, Germany) with standardized parameters, including a uniform cement space. (Figure 4). Two types of 3D-printed resin materials were used to fabricate the veneer restorations through an additive manufacturing technique using a digital light processing (DLP) 3D printer (Asiga, Sydney, Australia), producing 3D-printed laminate veneers under standardized conditions (fixed printing angle, resolution, and post-curing protocol) to ensure consistency, as illustrated in (Figure 4).

The sample teeth were randomly divided into 8 groups of 10 samples each. The final sample grouping, after having received two types of 3D-printed resin veneers, was as follows:

- Restored incisor restored with Temp Print veneer (RIT);
- Restored incisor restored with Varseosmile Triniq veneer (RIV);
- Restored canine restored with Temp Print veneer (RCT);
- Restored canine restored with Varseosmile Triniq veneer (RCV);
- Sound incisor restored with Temp Print veneer (SIT);
- Sound incisor restored with Varseosmile Triniq veneer (SIV);
- Sound canine restored with Temp Print veneer (SCT);
- Sound canine restored with Varseosmile Triniq veneer (SCV).

### 2.7. Surface Treatment and Cementation Protocol

**Figure 5:**
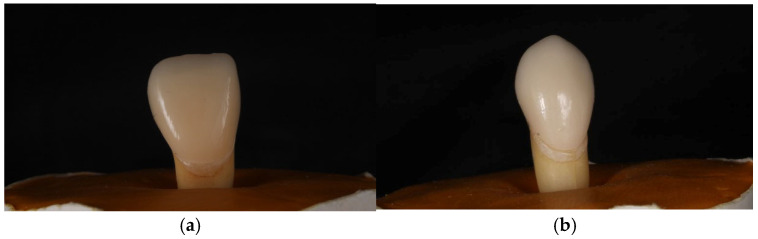
3D-printed veneer restorations: (**a**) Incisor tooth restored with Varseosmile Triniq 3D-printed veneer restoration; (**b**) canine tooth restored with Temp Print 3D-printed veneer restoration.

Each sample surface of the 3D-printed restoration underwent a gently sandblasting with aluminum oxide at 2.0 bar. Following this, the surfaces were cleaned using oil-free and water-free compressed air. An adhesive-enhancing primer (GC Corporation, Tokyo, Japan) was applied to the inner surfaces and subsequently dried. The teeth were cleaned; the enamel was etched, then rinsed and dried before bonding. 10-MDP-based universal adhesive bond (GC Corporation, Tokyo, Japan) was vigorously rubbed in the enamel surface and then air-blown at high pressure for 5 s without light curing. The surface of the pre-existing composite in restored teeth was roughened using a fine-grit diamond bur and was also cleaned, etched, and treated with the adhesive system. Veneers were cemented using a dual-cure resin cement (GC Corporation, Tokyo, Japan). The cement was put on the inner surface of the 3D-printed veneer, which was then pressed onto the teeth under controlled pressure. Excess cement was removed after light curing for a short time (3–4 s), then removed with the tip of an explorer under magnification loupes (Carl Zeiss AG, Oberkochen, Germany), and finally light cured with a curing light unit (B.A. International Ltd., Northampton, UK). Each wall surface of the restoration received light curing for 60 s to enhance polymerization, followed by finishing the margins with flexible disks (3M ESPE, St. Paul, MN, USA) (Figure 5).

### 2.8. Microleakage, Marginal Fitness, Cement Voids, Cement Loss Evaluation

All restored teeth with 3D-printed veneers were coated with nail varnish, exposing a 1 mm area around the adhesive interface on the cervical and proximal walls. A diluted silver nitrate solution (1:4 silver nitrate to distilled water) was prepared, filtered, and placed in a syringe for testing. Each tooth specimen was immersed in this solution under laboratory lighting for 24 h and subsequently rinsed three times with water for over 30 min. The varnished teeth were cleaned with acetone before being soaked in a diluted photo developer solution (Afga Dntus D-1000, Afga NV, Septestraat 27, Mortsel, Belgium; 4.5:1.0 ratio of distilled water to photo developer solution) for 8 h, followed by another three rinses with water. All the teeth were embedded in transparent self-curing acrylic resin blocks (Ivoclar Vivadent AG, Schaan, Liechtenstein) to facilitate handling and standardization during preparation and evaluation. The teeth were positioned so that the cemento-enamel junction (CEJ) level is close to the acrylic surface to ensure measurement stability. The teeth were sliced with a low-speed diamond saw under water cooling into three to four 1 mm thick slices along their long axis through the cervical margin and perpendicular to the proximal margin. These slices were examined with a digital stereomicroscope at magnifications of 2×, 4×, 6×, and 10. These procedures were conducted based on previous studies [18].

Microleakage evaluation:

Microleakage evaluation was conducted based on previous studies [18]. Two observers separately assessed the quantity of tracer along the veneer interface by using the scheme as follows.

0 = no microleakage in the veneer restoration interface.

1 = 0% to 20% of the veneer restoration interface showing microleakage.

2 = 20% to 40% of the veneer restoration interface showing microleakage.

3 = 40% to 60% of the veneer restoration interface showing microleakage.

4 = 60% to 80% of the veneer restoration interface showing microleakage.

5 = 80% to 100% of the veneer restoration interface showing microleakage.

Additionally, marginal fitness, cement voids and loss were evaluated under microscope and magnification loupes, following the methodologies previously described in the literature for assessing marginal adaptation and interfacial defects in dental restorations [19,20,21,22], as described below:

Cement Void Assessment:

Cement voids were assessed by two independent observers who separately assessed the presence or absence of voids (air bubbles or gaps) at the tooth–veneer restoration interface along both longitudinal and cross-sectional views of the examined samples. The findings were recorded as scores to be analyzed statistically as follows:

- Presence of cement void (score 1) = interfaces showing cement voids.
- Absence of cement void (score 0) = interfaces showing no cement voids.

Cement Loss Assessment:

Cement loss was also evaluated by the same two observers, who independently assessed the presence or absence of cement loss at the tooth–veneer restoration interface along the longitudinal and cross-sectional views of the examined samples. The observations were recorded as scores to be analyzed statistically as follows:

- Presence of cement loss (score 1) = interfaces showing cement loss.
- Absence of cement loss (score 0) = interfaces showing no cement loss.

**Figure 6:**
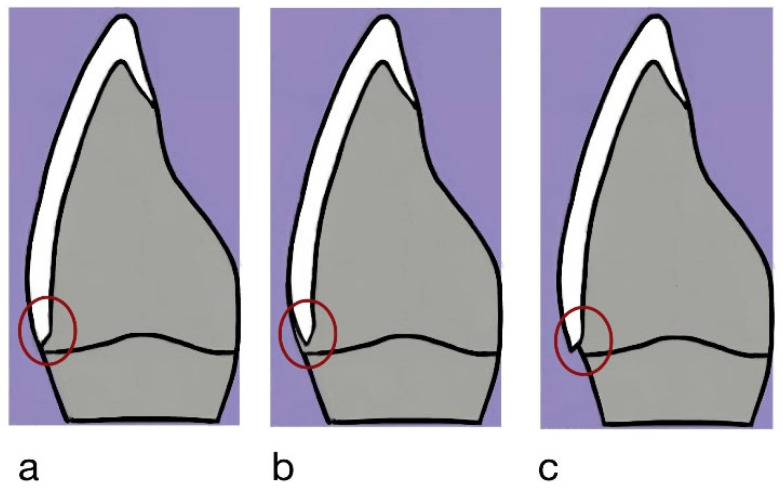
Schematic representation of marginal fitness evaluation: (**a**) (Score 0) veneer margin coinciding with the finish line; (**b**) (score 1) veneer margin located below the finish line; (**c**) (score 2) veneer margin extending beyond the finish line. The red circle shows veneer margin location.

Marginal Fitness Evaluation:

Marginal fitness evaluation was done according to the same procedure mentioned previously in the evaluation of cement voids and loss. The veneer margin was assessed in relation to the prepared finish line and categorized as overextended, underextended, or well-fitted, as illustrated in (Figure 6). The recorded scores were assigned as follows:

- Score 0: Veneer margin coinciding with the finish line (ideal fit).
- Score 1: Veneer margin located below the finish line (underextension).
- Score 2: Veneer margin extending beyond the finish line (overextension).

Two independent, blinded examiners evaluated all specimens. Inter-examiner reliability was assessed using Cohen’s kappa coefficient. The inter-examiner reliability for microleakage, measured as an intraclass correlation coefficient (ICC), was 0.850, while the kappa values for cement loss, cement void and marginal fitness were 0.855, 0.834 and 0.865, respectively.

### 2.9. Ethics Statement

The study was performed in accordance with the ethical standards of the Institutional and National Research Committee and the Declaration of Helsinki of 1964, and its later amendments or comparable ethical standards, regarding extracted human teeth for in vitro experimental purposes only. The Committee for Clinical Ethics (Azienda Ospedaliero-Universitaria Senese) approved the use and conservation of the samples and the procedure for informed consent, and issued a positive opinion in its session on 17 January 2025, Code no. 19/2024. The extracted teeth were used for research purposes after informing the patients and obtaining their consent. The study did not involve the direct participation of patients or any intervention on patients.

### 2.10. Statistical Analysis

Data description, analysis, and presentation were analyzed using the Statistical software for Social Science (SPSS version 22, Chicago, IL, USA). The microleakage test used the Wilcoxon sum rank test, with descriptive statistics, such as minimum, maximum, median, and mean rank. Other variables, such as cement void, cement loss, and marginal fitness, were analyzed using Fisher’s exact test, with the frequency and percentage as descriptive statistics. The level of significance was set at *p* < 0.05. Additionally, the sample size was calculated using G*Power (version 3.1.9.7; developed by Franz Faul, University of Kiel, Germany). Based on a two-tailed test, an alpha error probability of 0.05, a statistical power of 95%, and a large effect size of Cohen’s D of 0.85, a sample size of 80 was determined. To ensure observer reliability, inter-examiner reliability was assessed using Cohen’s kappa coefficient. The inter-examiner reliability for microleakage, as an intraclass correlation coefficient (ICC), was 0.850, while the kappa values for cement loss, cement void and marginal fitness were 0.855, 0.834 and 0.865, respectively.

## 3. Results

### 3.1. Microleakage Test

Microleakage scores were mainly 0 across all tested groups at both proximal and cervical surfaces. In restored incisors, both Temp Print and Varseosmile Triniq groups demonstrated a median microleakage score of 0 at the proximal surface (mean rank (MR) = 5.50 for both; *p* = 1.000). At the cervical surface, the median remained 0 for both materials (MR = 5.00 vs. 6.00; *p* = 0.690), with only one isolated high score (score = 5) observed in the Varseosmile subgroup. In restored canines, the proximal surface also showed a median of 0 for both materials (MR = 5.00 vs. 6.00; *p* = 0.690). Cervically, median values remained 0 (MR = 4.50 vs. 6.50; *p* = 0.310), with only occasional higher scores recorded. For sound teeth, minimal deviations from score 0 were observed. In sound incisors, proximal median values were 0 for both materials (MR = 4.00 vs. 7.00; *p* = 0.151), while cervical results also showed a median of 0 (MR = 5.00 vs. 6.00; *p* = 0.690). Sound canines demonstrated similar findings, with proximal median = 0 (MR = 6.50 vs. 4.50; *p* = 0.310) and cervical median = 0 (MR = 6.00 vs. 5.00; *p* = 0.690), as illustrated in Table 1 and Table 2.

**Figure 7:**
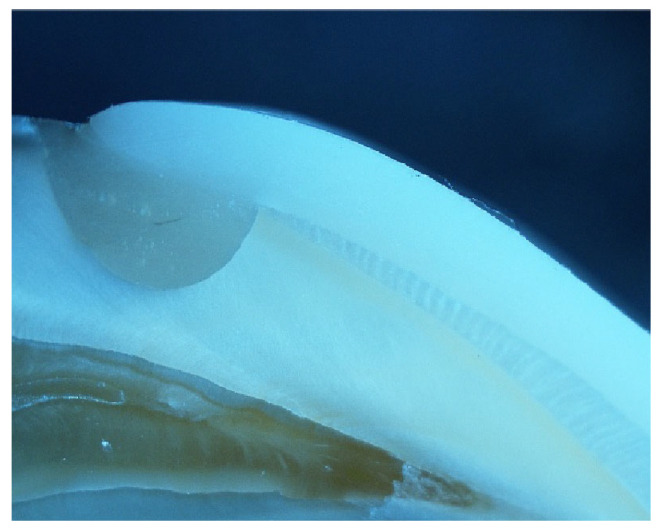
Microscopic image of a representative specimen from a tested group shows a microleakage score of 0 at the cervical surface (×4).

**Figure 8:**
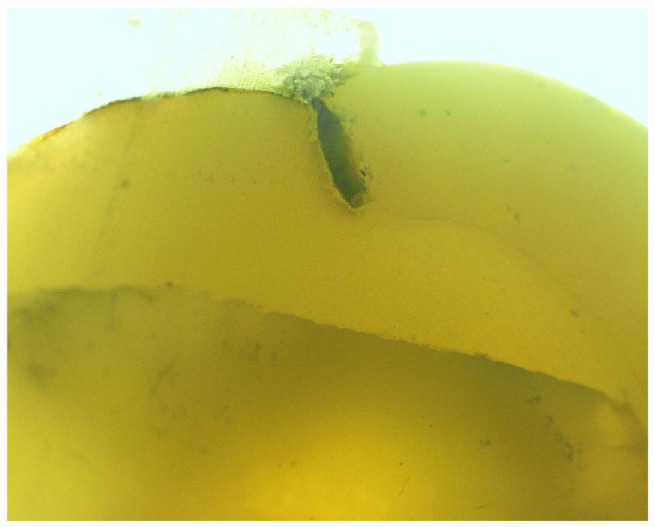
Microscopic image of a representative specimen from a tested group with a microleakage score of 5 at the proximal surface (×6).

Comparison between proximal and cervical surfaces revealed no statistically significant differences across all subgroups (*p* ≥ 0.310). Overall, no statistically significant differences were detected between veneer materials, tooth conditions, or tooth types (all *p* > 0.05), indicating comparable microleakage performance and suggesting that any observed variations were likely due to random variation rather than a true material or condition effect, as shown in Table 3 and (Figure 7 and Figure 8).

### 3.2. Marginal Fitness Test

Marginal fitness results were expressed as frequency distributions (fit, over, under) and analyzed using Fisher’s exact test. At the proximal surface, restored incisors showed 60% fit for Temp Print and 100% fit for Varseosmile Triniq (*p* = 0.444). Sound incisors demonstrated 20% fit for Temp Print and 60% fit for Varseosmile (*p* = 0.524). In canines, both materials showed identical distributions in sound teeth (40% fit each; *p* = 0.999), while restored canines showed 80% fit for Temp Print and 100% for Varseosmile (*p* = 0.999), as illustrated in Table 4.

At the cervical surface, sound incisors showed 80% fit for Temp Print and 100% for Varseosmile (*p* = 0.999), while restored incisors demonstrated 40% fit for both materials, with additional under- and over-extensions observed in the Varseosmile group (*p* = 0.999). Sound canines demonstrated 100% fit for both 3D-printed materials, whereas restored canines showed 60% fit for Temp Print and 20% for Varseosmile (*p* = 0.526), as illustrated in Table 5.

Despite some numerical differences in distribution, statistical analysis showed no significant difference between veneer materials, tooth types and tooth conditions on both proximal and cervical surfaces (all *p* ≥ 0.444). These results show that there was no significant difference in marginal adaptation between all tested groups, and the observed differences are likely due to random distribution rather than systematic differences.

### 3.3. Cement Void Test

Almost all groups had no cement voids. In restored incisors, only one sample at the proximal surface and two samples at the cervical surface in the Temp Print subgroup exhibited cement voids. Restored canines showed no cement voids at either surface for both materials.

**Figure 9:**
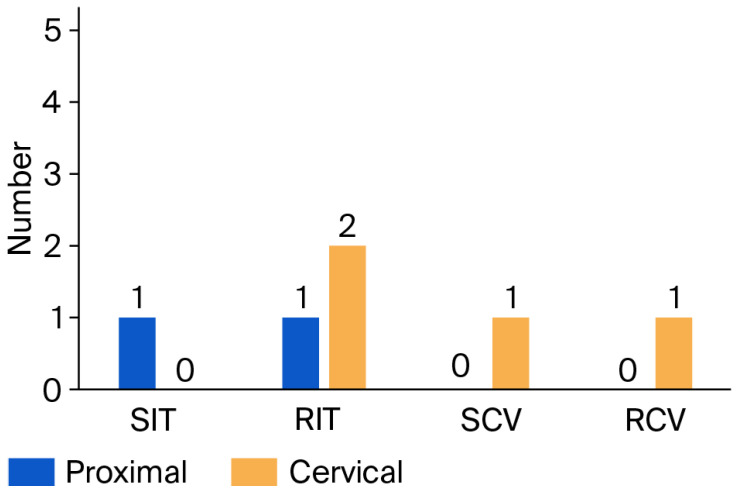
Chart of proximal and cervical cement void among veneer materials.

Only one sample in the Temp Print subgroup (proximal, incisors) and one sample in the Varseosmile subgroup (cervical, canines) showed cement voids in sound teeth, whereas all other samples were void-free. Statistical analysis showed no significant differences between veneer materials, tooth types, or tooth conditions at both proximal and cervical surfaces (*p* = 0.999). This indicates that cement-void occurrence was minimal and comparable between all tested groups, without a material-dependent effect, as presented in Figure 9.

### 3.4. Cement Loss Test

Cement loss was infrequent in all groups. Restored incisors and canines showed no cement loss at the proximal surface for either material. At the cervical surface, only one restored canine sample in the Varseosmile subgroup exhibited cement loss.

**Figure 10:**
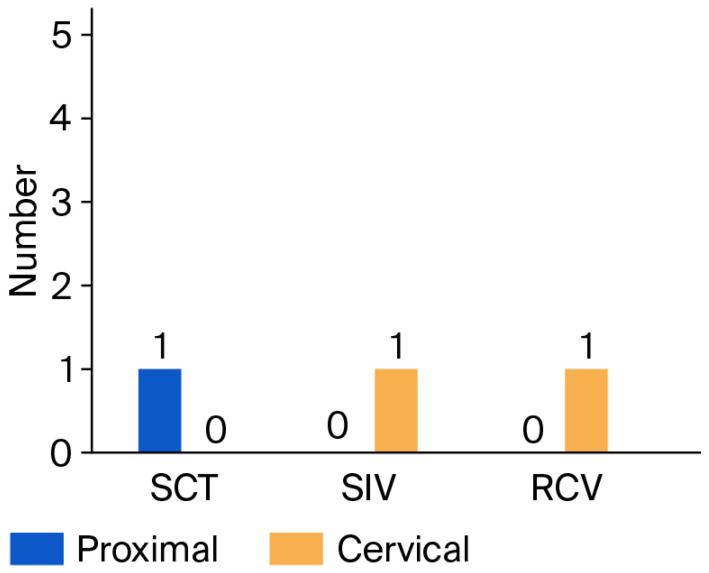
Chart of proximal and cervical cement loss among veneer materials.

No cement loss was found in incisors of sound teeth at both surfaces, except for one cervical sample in the Varseosmile subgroup. Only one proximal sample in the Temp Print subgroup of sound canines presented cement loss, while all cervical samples were free of cement loss. Cement loss was not statistically different between veneer materials, tooth type and tooth condition at proximal and cervical surfaces (*p* = 0.999). This shows that the cement loss was minimal and not statistically different between the tested variables, indicating similar clinical performance, as illustrated in Figure 10.

## 4. Discussion

The assessment and the integrity of the marginal seal, durability and biocompatibility are crucial factors to the clinical success of veneer restorations in an oral environment, particularly with the increasing adoption of 3D-printing technologies [23]. Due to differences in the physical properties of tooth substrates and restorative materials, achieving optimal marginal adaptation remains a challenging issue. Therefore, evaluating microleakage, marginal fitness, cement void and cement loss is essential for assessing the laboratory and clinical reliability of novel restorative materials. This study assessed two 3D-printed resin materials (GC Temp Print and Varseosmile Triniq) across different tooth types (incisors and canines) and conditions (sound and restored). The results consistently show no statistically significant differences among all examined groups (*p* > 0.05).

The similarity in material composition and polymerization mechanisms could be an explanation for the absence of significant differences. Both materials are resin-based systems, light-cured and DLP-fabricated and designed to provide high dimensional accuracy and stability. In addition, bonding procedures, cementation protocols and the standardization of preparation design likely minimized experimental variability, thereby reducing the likelihood of detecting intergroup differences.

Microleakage findings revealed that most samples exhibit a score of 0, demonstrating effective marginal sealing regardless of 3D-printed material, tooth type, or condition. The lack of statistically significant differences (*p* > 0.05) suggests that neither material demonstrated superior sealing ability. This may be attributed to effective adhesive bonding and similar behavior of polymerization shrinkage for both of them. These results are in agreement with previous studies reporting that properly processed 3D-printed resin materials can achieve marginal integrity comparable to conventionally fabricated restorations [23,24].

Marginal fitness results showed no significant differences despite some variation in categorical distribution (fit, over, under). This may reflect the potential precision of digital workflow, where additive manufacturing and CAD software design reduce operator-dependent variability. The fabrication technique plays a more critical role than tooth type in determining marginal adaptation, as emphasized by previous studies [25]. The type of 3D-printing technology and building angle significantly affect the precision and marginal fit of restorations [24]. Restorations produced through 3D printing often exhibit improved volumetric interfacial adaptability [26].

The lower occurrence of cement voids and cement loss, with no statistically significant differences between the examined groups (*p* = 0.999), indicates that the cementation protocol was dependable, reliable and consistent. Factors such as the printing orientation of (45°), proper and sufficient post-curing process, and surface treatment, such as air abrasion, likely contributed to improved micromechanical retention and wettability, thereby reducing interfacial defects of restorations [27,28]. Additionally, standardized digital design parameters of cement space may have contributed to uniform cement thickness, thus minimizing areas susceptible to void formation or dissolution [29].

Several studies have reported that in the fabrication of 3D-printed dental resin materials, the commonly used printing angulation is 45°, as it provides a good balance between printing accuracy and mechanical performance. In this study, the printing angulation used for the samples was 45°. Recent studies have demonstrated that this angulation can enhance mechanical properties by improving layer adhesion and stress distribution [30,31].

Importantly, no differences were observed between sound and restored teeth, suggesting that the adhesive interface provided by the bonding system was effective in both cases. This finding supports previous reports indicating that the substrate type (enamel or composite) had a limited effect on marginal adaptation when reliable adhesive protocols are correctly implemented [25]. This is clinically important, as veneers are frequently placed on previously restored teeth, and concerns regarding compatibility at the adhesive interface remain a topic of ongoing investigation.

The results of this study suggest that the bond to a previously restored composite surface is effective. Modern universal adhesives containing monomers like 10-MDP create a strong chemical bond with the organic matrix of both the composite restoration and the new 3D-printed resin [32].

Similarly, no significant differences were identified between incisors and canines or between proximal and cervical margins. Although cervical margins are traditionally associated with higher microleakage risk due to bonding challenges in dentin or cementum, as well as different anatomical landmarks between cervical and proximal aspects, the current results suggest that modern adhesive systems may mitigate these limitations under controlled conditions [33,34]. The fact that the restorations did not influence the marginal adaptation should be stressed, in that fresh, new restorations must be made clinically and then sandblasted and silanized, as was done in this in vitro study, before preparing laminates. However, it is advised to never leave old, uncertain, and unknown composite restorations [35].

The accuracy of digital impressions and margin design also plays an important role in marginal adaptation. Previous studies have shown that scanner trueness is maintained across different materials as long as the finish line is smooth and well-defined, allowing CAD software to accurately detect and reproduce restoration margins [35,36]. In the present study, careful preparation of a continuous and polished finish line was conducted. This approach likely contributed to reducing the potential for step or ledge artifacts that can occasionally occur at the margins of a restoration.

The choice of 3D-printed resin and manufacturing parameters may influence marginal accuracy. Ceramic-filled resins such as GC temp print and Varseosmile triniq are designed to provide dimensional stability and reduced polymerization shrinkage. Additive manufacturing techniques may offer improved internal adaptation compared to subtractive methods by minimizing milling-related inaccuracies [27,36]. Furthermore, proper finishing and polishing procedures, as well as surface preparation protocols, are essential to optimize marginal adaptation and bonding performance [37].

Cement void and cement loss outcomes are influenced by different factors, including surface treatment, degree of polymerization and marginal fit. Recent research highlights that the longevity of the cement interface is influenced by the precision of the 3D printing process, the specific surface treatments applied to the resin, and the resulting marginal fit [27]. Inadequate post-curing of 3D-printed restoration may lead to insufficient polymer conversion, leaving residual monomers that compromise bonding effectiveness and increase the risk of cement degradation [29]. Nevertheless, when post-curing protocols are optimized, the performance of 3D-printed materials can improve significantly. Extended post-curing enhances the mechanical properties and marginal sealing of printed crowns, indicating that proper polymer network formation plays a key role in minimizing microleakage. Sandblasting of the intaglio surface as a type of appropriate surface treatment could enhance micromechanical retention and contribute to the stability of the adhesive interface [27].

The absence of significant differences in cement loss may also be attributed to or related to the high precision of 3D-printing (DLP technology) shared by both materials. Previous studies indicate that 3D-printed veneers can achieve marginal gaps as low as 56–79 µm, which fall within clinically acceptable limits [38,39]. When the marginal fit is consistently within this clinically acceptable range across all groups, the cement layer thickness remains uniform. Digital software allows for a standardized cement space (typically 30–50 µm). This uniformity minimizes the volume of exposed cement at the margins, which is the primary site for cement loss [34].

From a clinical point of view, the lack of statistically significant differences (*p* > 0.05) could be understood as evidence of similar or comparable performance. This suggests that both materials may be used without a measurable difference in marginal integrity or cementation quality. However, it is important to recognize that a non-significant result indicates that observed differences fall within the range of random variation.

GC Temp Print has been marketed for long-term temporary restorations, while Varseosmile Triniq is intended for permanent restorations; their immediate and mid-term marginal performance may be comparable. These 3D-printed materials have a nanohybrid filler technology to minimize polymerization shrinkage. Minimal shrinkage during the printing and post-curing stages guarantees that the veneer remains unwrapped, hence preventing stress on the cement [40]. However, recent articles showed very good clinical short-term performances of restorations made with one of the two tested resins [11,39].

The hybrid substrate (enamel and composite) does not inherently predispose the restoration to premature debonding as long as the margins of the veneer are well-supported and the bonding protocol is strictly followed. This is because the elasticity of these resins (usually ranging between 3000 and 4500 MPa) is closer to that of the underlying dentin and composite complex than traditional ceramics [36].

The absence of significant differences between the groups studied may also be explained by the strict standardization of the experimental variables. In addition, the size and geometry of Class III and Class V cavity preparations were standardized. Further studies are needed to evaluate factors such as saliva contamination, occlusal loading, and aging that may affect the long-term performance of veneer restorations in clinical conditions. However, it is acknowledged that microleakage and marginal integrity are time-dependent phenomena. Additionally, the results cannot be directly generalized to other veneer materials, such as lithium disilicate (E-max), as the type of veneer restoration may affect the tested parameters.

The limitations of this study are essentially the in vitro conditions, the limited number of sample teeth and the limited number of tested 3D-printed materials. The evaluation of a wider number of samples in each experimental group and of 3D-printed resins is desirable, and a multicenter randomized controlled trial to verify lab results under real clinical conditions is advocated.

## 5. Conclusions

Pre-existing Class III and Class V composite restorations did not negatively influence the marginal integrity, microleakage, or overall sealing ability of 3D-printed resin veneers.

Restored abutments with composite resin in proximal and cervical surfaces can successfully receive a 3D-printed veneer restoration and still have a comparable marginal integrity regarding microleakage, marginal fitness, cement void and cement loss with sound abutments. Both tested 3D-printed resin materials demonstrated good marginal sealing, suggesting their suitability for use on both sound and restored anterior teeth.

## Figures and Tables

**Table 1:** Descriptive and statistical test of proximal microleakage among veneer materials, using Wilcoxon sum rank test.

		Veneer Material								W	*p*-Value
		Temp Print				Varseosmile					
		Min.	Max.	Median	MR	Min.	Max.	Median	MR		
Incisor	Sound	0	0	0	4.00	0	5	1	7.00	20.0	0.151
	Restored	0	0	0	5.50	0	0	0	5.50	27.5	1.000
Canine	Sound	0	1	0	6.50	0	0	0	4.50	22.5	0.310
	Restored	0	0	0	5.00	0	5	0	6.00	25.0	0.690

**Table 2:** Descriptive and statistical test of cervical microleakage among veneer materials, using Wilcoxon sum rank test.

		Temp Print				Varseosmile				W	*p*-Value
		Min.	Max.	Median	MR	Min.	Max.	Median	MR		
Incisor	Sound	0	0	0	5.00	0	1	0	6.00	25	0.690
	Restored	0	0	0	5.00	0	5	0	6.00	25	0.690
Canine	Sound	0	5	0	6.00	0	0	0	5.00	25	0.690
	Restored	0	0	0	4.50	0	5	0	6.50	22.5	0.310

**Table 3:** Descriptive and statistical test of microleakage between cervical and proximal surfaces, using the Wilcoxon rank sum test.

			Proximal	Cervical	W	*p*-Value
			Mean Rank	Mean Rank		
Incisor	Sound	Temp print	5.50	5.50	27.50	1.000
		Varseosmile	6.60	4.40	22.00	0.310
	Restored	Temp print	5.50	5.50	27.50	1.000
		Varseosmile	5.00	6.00	25.00	0.690
Canine	Sound	Temp print	5.80	5.20	26.00	0.841
		Varseosmile	5.50	5.50	27.50	1.000
	Restored	Temp Print	5.50	5.50	27.50	1.000
		Varseosmile	5.10	5.90	25.50	0.690

**Table 4:** Descriptive and statistical tests of proximal marginal fitness among veneer materials, using Fisher’s exact test to assess proximal fitness.

Teeth	Tooth Condition	Veneer Material	Fit	Over	Fisher’s Exact
			N. (%)	N. (%)	
Incisor	Sound	Temp Print	2 (20)	8 (80)	0.524
		Varseosmile	6 (60)	4 (40)	
	Restored	Temp Print	6 (60)	4 (40)	0.444
		Varseosmile	10 (100)	0 (0)	
Canine	Sound	Temp Print	4 (40)	6 (60)	0.999
		Varseosmile	4 (40)	6 (60)	
	Restored	Temp Print	8 (80)	2 (20)	0.999
		Varseosmile	10 (100)	0 (0)	

**Table 5:** Descriptive and statistical test of cervical marginal fitness among veneer materials, using Fisher’s exact test to assess cervical fitness.

Teeth	Tooth Condition	Veneer Material	Fit	Under	Over	Fisher’s Exact
			N. (%)	N. (%)	N. (%)	
Incisor	Sound	Temp Print	8 (80)	2 (20)	0 (0)	0.999
		Varseosmile	10 (100)	0 (0)	0 (0)	
	Restored	Temp Print	4 (40)	6 (60)	0 (0)	0.999
		Varseosmile	4 (40)	4 (40)	2 (20)	
Canine	Sound	Temp Print	10 (100)	0 (0)	0 (0)	----
		Varseosmile	10 (100)	0 (0)	0 (0)	
	Restored	Temp Print	6 (60)	4 (40)	0 (0)	0.526
		Varseosmile	2 (20)	6 (60)	2 (20)	

## Data Availability

The data presented in this study are available upon request from the corresponding author. The data are not publicly available due to the University of Siena’s policy.
